# Development and Characterization of Astaxanthin-Containing Whey Protein-Based Nanoparticles

**DOI:** 10.3390/md17110627

**Published:** 2019-11-04

**Authors:** Francesca Zanoni, Martina Vakarelova, Gianni Zoccatelli

**Affiliations:** 1 Department of Biotechnology, University of Verona, Strada Le Grazie 15, 37134 Verona, Italy; francesca.zanoni@univr.it; 2 Sphera Encapsulation S.r.l., Strada Le Grazie 15, 37134 Verona, Italy; vakarelova@spheraencapsulation.com

**Keywords:** encapsulation, astaxanthin, *Haematococcus pluvialis*, nanoparticles, bioaccessibility, stability, simulated digestion

## Abstract

Astaxanthin (ASX) is a carotenoid of great interest due to its potential health benefits. However, its use in the food, feed, and pharmaceutical fields is limited due to low bioavailability, poor stability during thermochemical treatments, susceptibility to oxidation, and poor organoleptic characteristics. The aim of this work was to develop a method to stabilize astaxanthin extracted from the microalgae *Haematococcus pluvialis* (*H.p.*) and to improve its nutritional and functional properties through nanoencapsulation. Nanoparticles (NPs) were produced by emulsification–solvent evaporation technique starting from *H.p.* oleoresin using whey proteins concentrate (WPC) as stabilizer. The efficiency of encapsulation was 96%. The particle size (Z-average) was in the range of 80–130 nm and the superficial charge (measured as zeta-potential) was negative (−20 to −30 mV). The stability of the NPs upon resuspension in water was assayed through a panel of stress tests, i.e., extreme pH, UV radiation, Fe^3+^ exposition, and heating at 65 °C, that always showed a superior performance of encapsulated ASX in comparison to oleoresin, even if NPs tended to precipitate at pH 3.5–5.5. Simulated gastroenteric digestion was conducted to study the release of ASX in physiological conditions, and showed a maximum bioaccessibility of 76%, with 75% ASX converted into the more bioavailable free form. The collected data suggest that NPs might have possible future applications as supplements for human and animal diets.

## 1. Introduction

Astaxanthin (ASX) (3,3′-dihydroxy-β-β′-carotene-4,4′-dione) is a symmetric ketocarotenoid widely distributed in nature, responsible for the bright red to pink color of some crustaceans, salmon, and birds. This molecule attracted the interest of the scientific community thanks to its well-known antioxidant capacity, estimated as 10 times greater than other carotenoids, including lutein and β-carotene, and as 100 times higher than alpha tocopherol [1,2,3]. Furthermore, it has been proven to be effective against diabetes, cardiovascular disease, some type of cancers, gastric ulcers, and in skin protection against UV rays [4,5,6]. The major natural source of ASX is *Haematococcus pluvialis* (*H.p.*) a freshwater algae which under stress condition can accumulate up to 2%–5.3% of ASX based on dry weight [7,8].

The enrichment of food with ASX is limited due to its low stability to light, oxygen, high temperatures and extreme pH, which can affect its conjugated structure [9,10]. In addition, the strong lipophilic nature of ASX underlies its scarce bioavailability [11]. ASX is commercially available mainly in the forms of ASX oleoresin and *H.p.* aplanospores. Oleoresin is a dense oil which is difficult to manipulate. Furthermore, it does not meet consumer acceptance due to the algae-related taste and smell. Aplanospores represent the *H.p.* growth stage in which accumulated ASX reaches the maximum concentration. These cells are collected, dried, physically broken, and directly used as a supplement. Their rupture is necessary since aplanospores are characterized by three walls made of sporopollenin and algenan [12,13] that act as a natural barrier against oxidation and chemical attack [14], but also as an obstacle to human digestion and absorption [15].

Different technologies have been employed to reduce ASX degradation and to increase its bioavailability. These include microencapsulation by different approaches using a number of shell materials such as chitosan, alginate, pectin, maltodextrin, starch, etc. [10,16,17,18], but these structures are sometimes difficult to digest due to the lack of specific enzymes that can degrade the shell [10,16]. In some other cases, the micrometer range of the obtained capsules requires a mechanical disruption to release ASX. In another work, chitosan was used to protect ASX against oxidation with great success [10,19], but the insolubility in water and at neutral pH of chitosan and its scarce release properties render this solution unsuitable for many food applications. 

Nanoencapsulation represents an emerging strategy by which it is possible to improve the bioavailability of active molecules as well as their stability. Indeed, the incorporation of ASX and other carotenoids in colloidal systems with dimensions close to 100 nm or less could improve the cell uptake and, thus, the bioavailability. Some applications are represented by nanoemulsions and nanodispersions [11,20,21]. Other approaches described nanostructured lipid carriers (NLCs), solid lipid nanoparticles (SLN) [22,23], and polymeric nanospheres [24], but some limitations such as the modest drug loading [22] or the use of pure—hence expensive—astaxanthin could represent an obstacle to industrial applications [23,24].

In the present work, whey proteins concentrate (WPC), a generally recognized as safe (GRAS) material with high nutritional value, has been employed as a carrier to produce water-dispersible ASX nanoparticles (NPs). WPC represents an added-value byproduct of the cheese production chain and a cheaper solution in comparison to recently described solutions based on purified whey proteins [25]. The incorporation of carotenoids in a nanocarrier also poses relevant stability issues. Indeed, in comparison to the microencapsulated form, the higher surface to volume ratio can promote degradation rates [26]. For this reason, NP stability was assessed in different conditions of pH, in the presence of oxidant species, and upon UV light and heat treatment. The bioaccessibility, another critical issue common to all carotenoids, was studied by simulated gastroenteric digestion.

## 2. Results and Discussion

### 2.1. Encapsulation of Oleoresin

**Figure 1:**
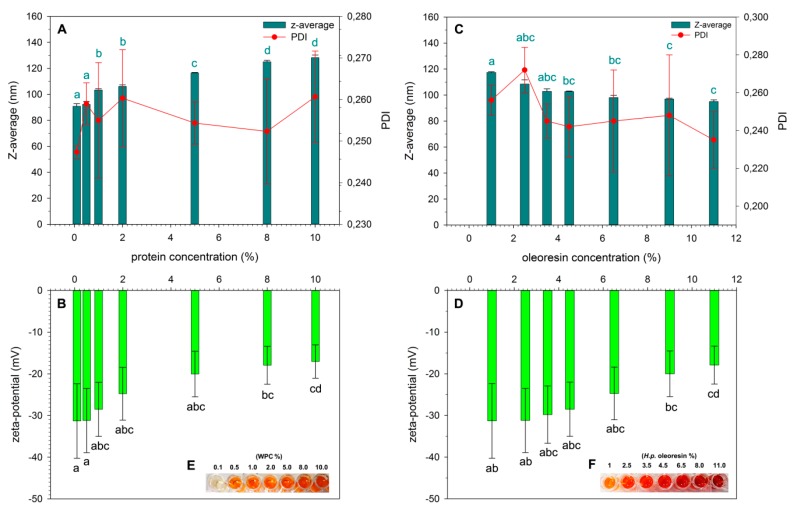
Variation of Z-average and PDI (**A** and **C**) and zeta-potential (**B** and **D**) as a function of protein concentration (**A** and **B**) and the *H.p.* oleoresin concentration (**C** and **D**) used to produce nanoparticles (NPs). Statistically significant differences (*p* < 0.05) between values are indicated by different letters. In panels (**A**) and (**C**), only the significance of the values relative to Z-average are indicated, since no differences for PDI were observed (*p* > 0.05). Appearance of the nanoparticles produced as function of protein concentration (**E**) and *H.p.* oleoresin concentration (**F**).

The encapsulation of *H.p.* oleoresin was carried out by an emulsification–solvent evaporation approach in which the lipophilic material is solubilized in ethyl acetate and emulsified with the water phase containing WPC, that acts as a stabilizer of oil-in-water interfaces. Two key parameters were evaluated to optimize the process. The first was the concentration of WPC. Size (Z-average), polydispersity index (PDI), and charge (zeta-potential) were monitored to study the role of protein concentration on the formation of the nanoparticles. As shown in Figure 1A, when keeping the oleoresin concentration at 1%, an increase in protein concentration led to an increase in NP diameter from an average size of 90 nm, obtained with the lowest concentration, to 128 nm with 10% WPC. PDI values were low for all the formulations, indicating the homogeneous size of the NP population, but no statistically significant differences were observed among the samples. These data apparently do not agree with those reported by Yi et al. [27] when changing the concentration of WPI from 0.1% to 1%, whereby they observed a halving in the mean particle diameter (from 190 to 90 nm) of encapsulated beta-carotene. The cause of this discrepancy can be due to a combination of different factors. Indeed, in our work, the lower concentration of lipophilic material considered was 10 times higher that of Yi et al. [27] (1% *H.p.* oleoresin vs. 0.1% beta-carotene). The oleoresin is actually a mixture of different lipophilic molecules, while the beta-carotene used by the authors was almost pure, suggesting that the two cores possess different physiochemical characteristics. In addition, the use of WPC (80% protein) in our work instead of whey protein isolate (WPI) (93% protein), with the former containing higher amounts of fats (~6% in WPC vs. ~1% in WPI), could have influenced the outcomes. 

Zeta-potential values of the nanoparticles were negative (Figure 1B) due to the whey protein shell that is negatively charged at neutral pH. Values higher than +20 and lower than −20 mV are normally associated to stable NPs [28] since, in this potential range, strong repulsive forces inhibit the natural aggregation phenomena-based hydrophobic and van der Waals interactions. The observed decrease in the magnitude of the zeta-potential by increasing protein concentration can be the result of the different extent of the unfolding of the proteins at interfaces, multilayer formation, or preferential adsorption of certain proteins as previously described [29,30]. Although the best values of Z-average, PDI, and zeta-potential were obtained with WPC concentrations ranging from 0.1% to 1%, a rapid degradation of ASX was experienced at concentrations from 0.1% to 0.5%. As a consequence, the following experiments were performed using 1% WPC concentration.

The second parameter considered was the concentration of *H.p.* oleoresin. As shown in Figure 1C, the diameter of the NPs slightly diminished with increasing oleoresin concentration. This result is in agreement with the previous data shown in Figure 1A, since the increase of oleoresin-to-protein ratio (WPC was kept at 1%) would diminish the multilayer aggregation of the proteins, decreasing the diameter of the particles. At the last point, obtained with 11% oleoresin, the main diameter reached 95 nm. In this case, the zeta-potential magnitude also decreased (Figure 1D), in agreement with the reduction of the diameter, reaching a surface charge higher than −20 mV (−17.9 ± 4.6 mV) at the last point. This value is normally associated with low stability of the nanostructure. This is possibly due to the high amount of oleoresin that might have exceed the capacity of the proteins to properly arrange on oil drop surface and thus influences the surface charge of the realized particles., In this case, the PDI values were also low and no significant differences were measured between the samples obtained by increasing oleoresin concentration. The appearance of the nanoparticles produced as a function of protein concentration and *H.p.* oleoresin concentration is respectively presented in panels E and F of Figure 1. Satisfactory results were given by the NPs produced with 4.5% *H.p.* oleoresin with a Z-average of 103 nm, a PDI of 0.242, and a surface charge of −28.5 mV. DLS results of the best solution (1% WPC, 4.5% *H.p.* oleoresin) are given in Supplementary Material. 

### 2.2. Characterization of NPs

**Figure 2:**
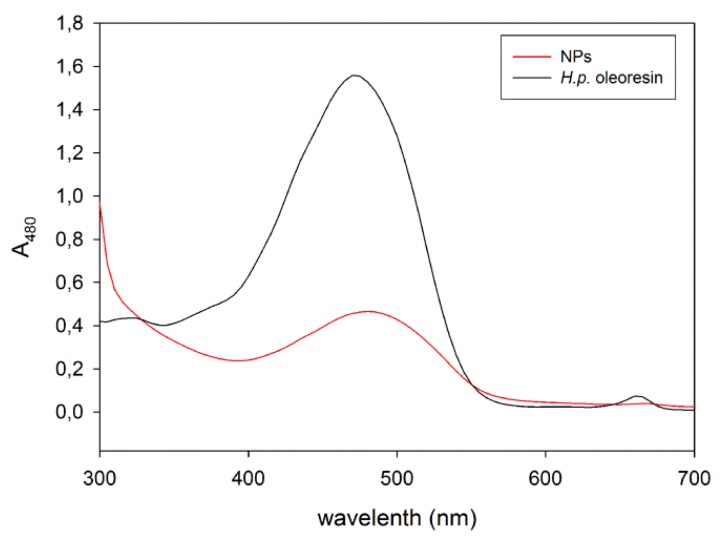
Comparison between the absorption spectra of the *H.p.* oleoresin and NPs.

A comparison between the absorption spectra of *H.p.* oleoresin in ethyl acetate and NPs in water revealed a redshift of the maximum absorption of ASX from 470 to 480 nm after the encapsulation process (Figure 2). This might be due to the presence of the protein shell together with the fact that the solvent was necessarily different. In the case of NPs, the great absorption at wavelengths < 300 nm is given by the presence of proteins. On the whole, the absorption characteristics of encapsulated astaxanthin in the visible spectrum are conserved. 

**Figure 3:**
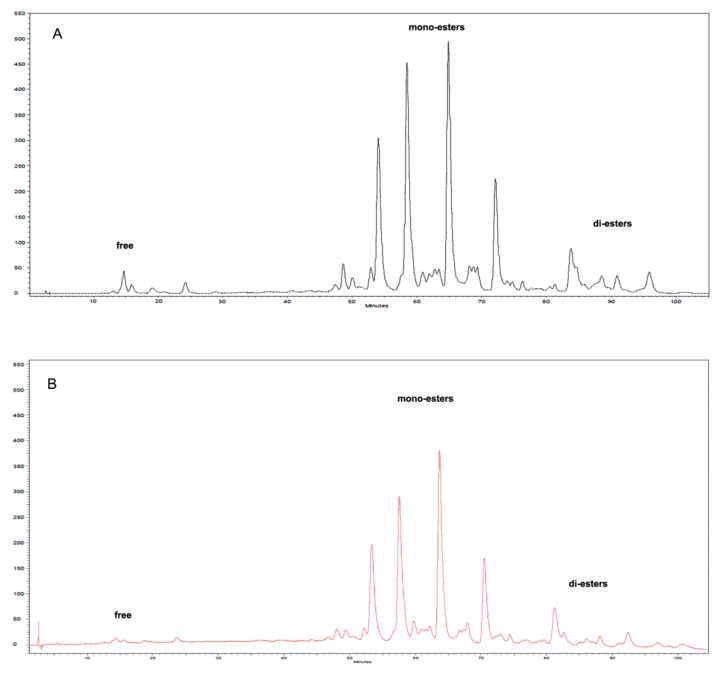
HPLC chromatograms of (**A**) *H.p.* oleoresin and (**B**) NPs extract composition showing free, monoesters and di-esters of astaxanthin (ASX) before and after encapsulation.

The encapsulation efficiency was 96.0% ± 2.5% with surface ASX accounting only for 0.16% ± 0.02%. The minor loss of ASX could be caused by the oxidation generated by the sonication process, or by the incomplete degradation of the protein shell during the enzymatic digestion that could limit the total solubilization of the carotenoid in the solvent. The HPLC analysis of the extract from optimized NPs was compared to the one of the *H.p.* oleoresin before the encapsulation process (Figure 3). No particular qualitative differences were observed, indicating that encapsulation process did not affect the nature of the esters’ distribution. 

Some works suggested that the radical scavenging activity (RSA) of ASX is mediated by the transfer of hydrogens or electrons, and in the case of the quenching of singlet oxygen, by energy transfer between the electrophilic singlet oxygen and the polyene chain [31]. ABTS represents one of the most used ways to evaluate the RSA of hydrophilic and highly lipophilic molecules such as carotenoids [32]. A concentration of 0.2 mg/mL of ASX from *H.p.* oleoresin was shown to have an RSA of 72.1%, while NPs, despite presenting 8 times lower astaxanthin concentration (i.e., 0.025 mg/mL vs. 0.2 mg/mL) exhibited an RSA of 95.8% (Table 1). The activity of WPC native proteins was tested and found to contribute up to 74.2% of the total NPs activity. This might be explained taking into account of the scavenging properties of some amino acid residues like cysteine, tyrosine, tryptophan, phenylalanine, and histidine present in the proteins structure [33].

### 2.3. Stability of the NPs

#### 2.3.1. Effect of pH

**Figure 4:**
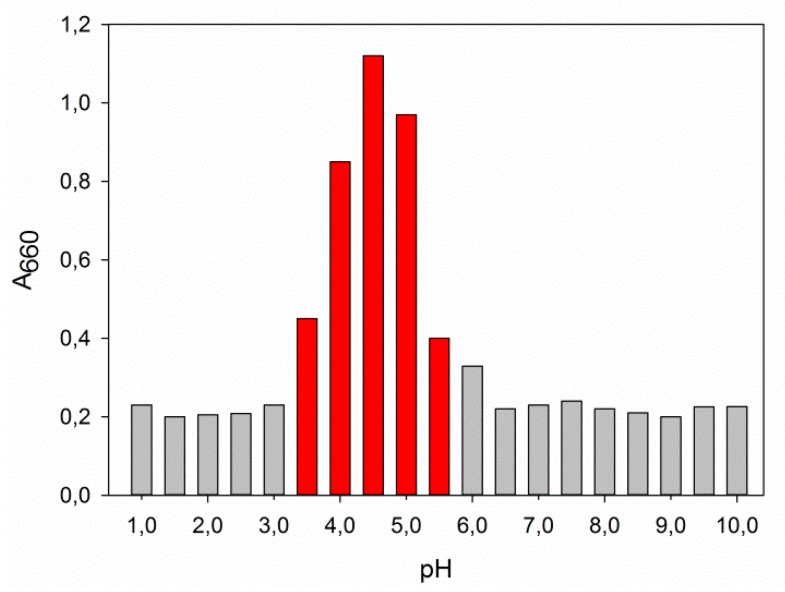
Stability of the NPs at different pH values expressed as turbidity measured spectrophotometrically at 660 nm.

NP stability was analyzed at different pH values. NPs were found unstable at pH between 3.5 and 5.5, giving the formation of agglomerates that tend to precipitate (Figure 4). The pH range corresponds to the average isoelectric point of the whey proteins. Qian and co-workers reported that the agglomeration of protein-stabilized nanoemulsions might originate from the small net surface charge registered at pH value close to the pI of the proteins, and thus not sufficient to exert electrostatic repulsion among the particles [34].

#### 2.3.2. UV Irradiation

**Figure 5:**
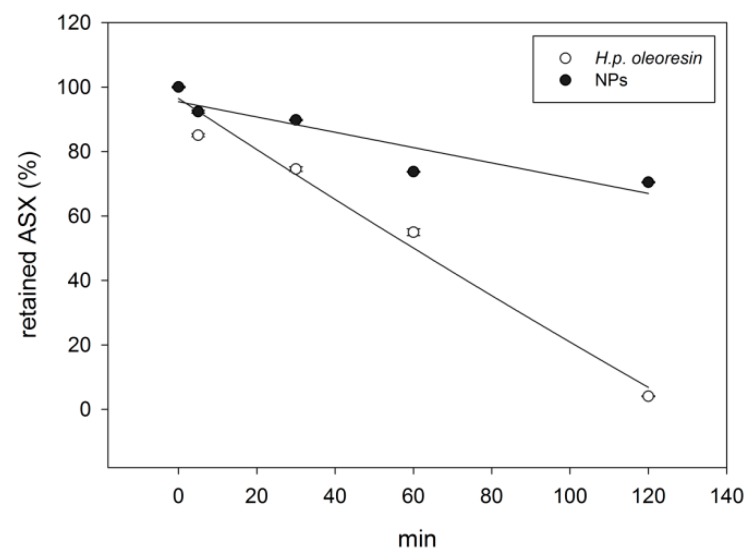
Comparison between ASX retained in NPs and in *H.p.* oleoresin after exposure to UV rays. The values are given as mean values ± standard deviation.

As already reported, one of the major factors responsible for the degradation of ASX is light [16,35,36]. Given the importance of this aspect the stability of NPs to UV irradiation was analyzed and compared to *H.p.* oleoresin. As shown in Figure 5, after two hours of exposure to UV-B light, the percentage of ASX in NPs and in *H.p.* oleoresin was 70.5% and 4.1% respectively, showing that the protein shell exerts a protective effect. In both cases, zero-order degradation kinetics were observable. This is not in agreement with previously reported studies [36] that described first-order degradation kinetics of ASX even though the wavelength of the emitting lamp was in UV-C range. This might have influenced the outcome. Indeed, in case of beta-carotene, the wavelength of the UV-lamp greatly influenced the degradation rate of the carotenoid [37].

#### 2.3.3. Fe(III)-Induced Oxidation

**Figure 6:**
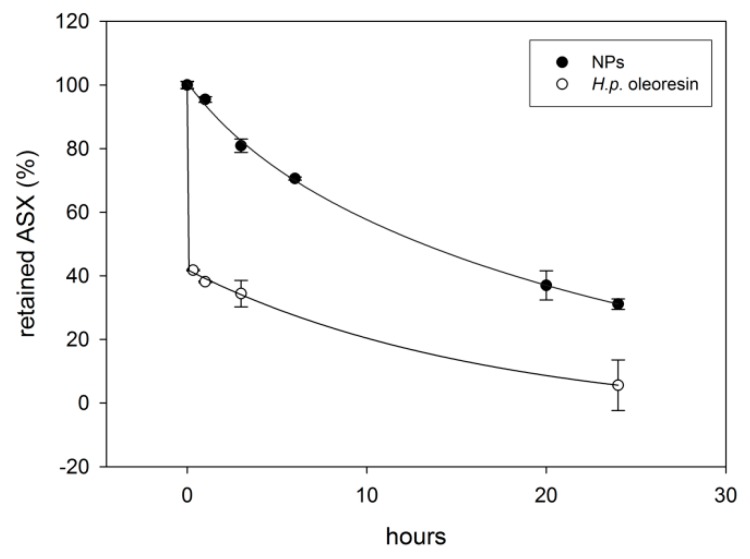
Comparison between ASX retained in NPs and *H.p.* oleoresin after exposure to FeCl_3_. The values are given as mean values ± standard deviation.

Another factor affecting ASX stability is the presence of pro-oxidant species. Many iron compounds that are ubiquitous in food products are known as harsh oxidizers. The physical barrier represented by the WPC shell and its intrinsic capacity to chelate metal ions could influence the stability of ASX contained in NPs [38,39]. To this purpose ferric chloride (FeCl_3_) is commonly used as an oxidizing agent to study carotenoid stability [38]. The results reported in Figure 6 show a different behavior between the samples: the *H.p.* oleoresin displayed a pattern characterized by a rapid degradation kinetics within the first 20 min of exposure with a loss of nearly 40% of the ASX content, followed by a slower rate of degradation until the end of the experiment with the retainment of only 5.6% ASX after 24 h. The NPs showed a slower decrease of ASX compared to the former. Indeed, after 20 min, the amount of ASX retained was 95%. After 24 h, the amount of ASX was 31%. The results showed a protective effect of the WPC protein shell towards Fe^3+^-mediated degradation. 

#### 2.3.4. Thermal Treatment

**Figure 7:**
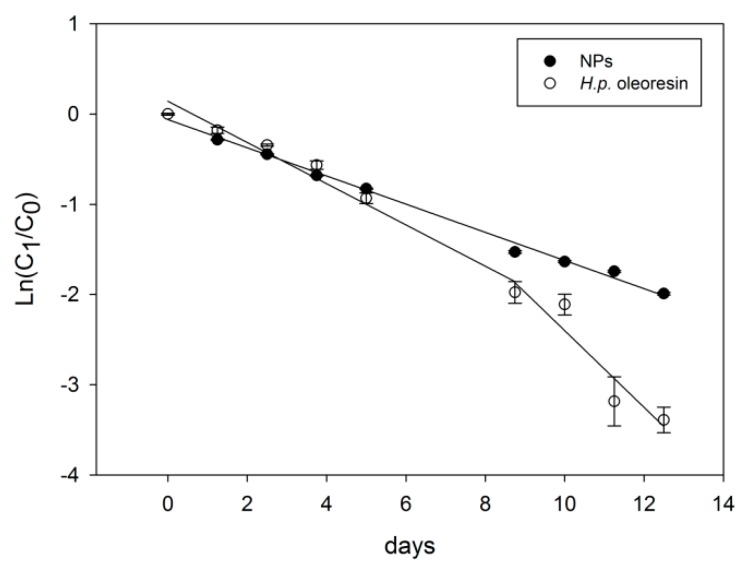
Degradation kinetics of NPs and *H.p.* oleoresin at 65 °C. The values are given as mean values ± standard deviation.

Accelerated tests [40] are regularly applied to study the stability of lipophilic substances such as edible oils. This test was employed to study the thermal stability of NPs and *H.p.* oleoresin (Figure 7). ASX present in NPs exhibited a typical first-order degradation kinetics, as already observed for many carotenoids [16,41,42]. On the contrary, within *H.p.* oleoresin, astaxanthin showed a profile characterized by two first-order kinetics: the first one with a lower reaction rate constant, close to that of the oleoresin, and the second one, starting from day 8, with a higher degradation rate. The quicker degradation of *H.p.* oleoresin could derive from the absence of the protective glassy matrix that allows for a faster accumulation of reactive degradation species [38,43] originating from the oxidation. When these degradation species reach a certain concentration, they could further oxidize the carotenoids present in *H.p.* oleoresin. A gradual loss of color was observed for both samples. As reported previously, the auto-oxidation products of carotenoids do not present color properties due to the lack of chromophores at the absorption wavelength of visible light [42,44]. HPLC analyses of ASX extracted from the NPs and the *H.p.* oleoresin showed the lack of a selective degradation of ASX: indeed, losses were observed for all the compounds present in the encapsulated *H.p.* oleoresin (data not shown). The absence of new peaks detected at 480 nm suggests the conversion of carotenoids into different products. It is reported that the thermal degradation of carotenoids in the presence of oxygen results in the formation of volatile compounds and larger non-volatile compounds [45]. The scarce protection of WPC shell against the thermal treatment could be due to the high surface exposed of the NPs, that could lead to a major exposure of ASX to heat and as a consequence to the degradation of the ASX carbon chain. 

### 2.4. Evaluation of Bioaccessibility by In Vitro Simulated Digestion

The absorption of carotenoids during digestion occurs mainly in the first part of the intestine. The lipophilicity of carotenoids is well known to limit their uptake, but in the case of ASX, the esterification with fatty acids represents another factor negatively affecting intestinal absorption, since esterified carotenoids are uptaken mainly as free form [46,47,48,49] 

**Figure 8:**
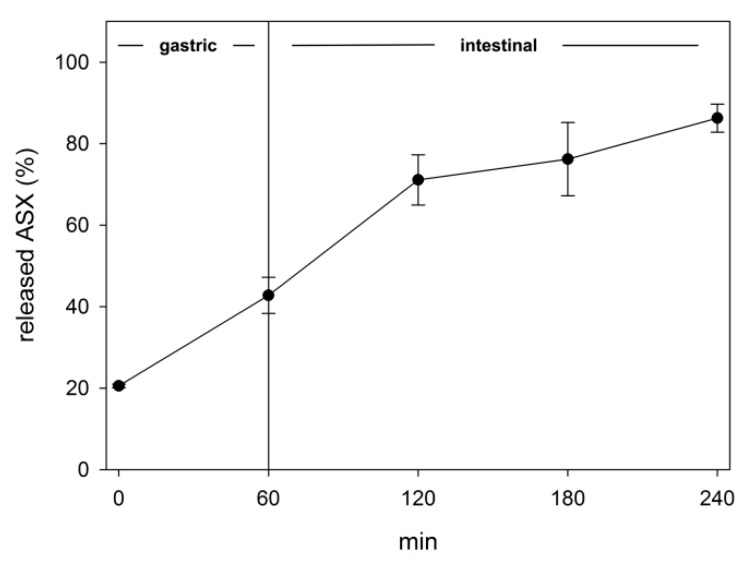
Release of ASX during *in vitro* simulated digestion of NPs. The values are given as mean values ± standard deviation.

Through simulated digestion (SD) experiments, it was possible to calculate that the amount of ASX that was released from NPs and, thus, bioaccessible, was about 43% after the gastric phase (Figure 8). This release was probably induced by a combination of two factors: (1) the activity of pepsin that partially degraded the whey protein shell, and (2) the low pH that can induce the agglomeration of the particles and the destabilization of the protein shell, as demonstrated by the release of 20% of the total ASX at time zero. Attempts to measure the particle size by DLS could not lead to reliable results due to high PDI values. 

During the intestinal phase, the agglomeration phenomena disappeared as a consequence of the neutralization of pH. This was confirmed also by the average size of the particles, 165.5 nm, and low PDI of 0.290. The release of ASX reached about 76% after 2 h of intestinal digestion that in this model, represents the small intestine [50,51]. After 3 h of intestinal digestion, the amount of bioaccessible ASX was 86%. 

**Figure 9:**
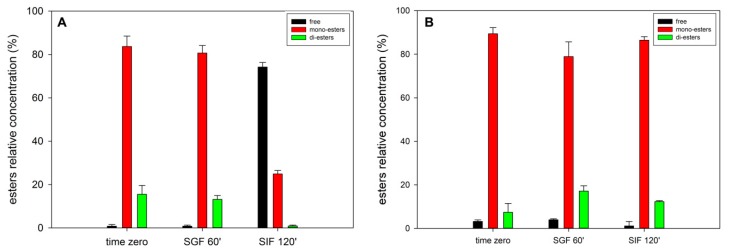
Relative concentration of the different ASX esterification forms at time zero and after 60 min digestion in SGF and 120 min digestion in SIF of NPs (**A**) and *H.p.* oleoresin (**B**).

The simulated digestion (SD) was performed not only to address the bioaccessibility of the encapsulated ASX but also to evaluate possible chemical modifications of the carotenoid. HPLC analysis of the oleoresin extracted from NPs collected before and after digestion could give a picture of the variation of the esterification degree of ASX. Figure 9 shows that the ester composition before digestion was mainly represented by monoesters and diesters accounting, on the whole, for 99% of the ASX present, while after two hours of intestinal digestion, the major form was represented by free ASX (75%). On the contrary, a sample of *H.p.* oleoresin diluted in soybean oil treated in the same way gave completely different results, i.e., the relative distribution of the esters was unaffected.

The combination of carotenoids with dietary fats and oils is reported to improve their bioaccessibility by facilitating the transfer to the micelle phase [52] and the micellarization process itself mediated by bile salts [53]. The difference observed between the two samples can be explained considering the dimension of the NPs. Indeed, the higher surface-to-volume ratio of the NPs could have promoted the greater hydrolysis of astaxanthin esters by lipase, the major enzyme involved in the hydrolysis of triacylglycerols [54]. Another explanation to the loss of hydrolysis in the sample containing *H.p.* oleoresin can be connected to the great amount of triacylglycerols present that, being the preferential substrate of lipase, might have hampered the activity of this enzyme towards other molecules such as astaxanthin esters.

This result is of great importance because, as mentioned above, the de-esterification of carotenoids is a crucial step required for their uptake through the intestinal mucosa.

Montero et al. [55] observed only 20% of total ASX in the free form upon digestion of a microencapsulated ASX system based on a gum arabic shell. The lower de-esterification can be explained considering different factors: (1) the difference in the matrix composition (whey protein vs. gum arabic), (2) the oleoresin to matrix ratio (1:1 in our case, 1:10 in Montero et al. [55], and (3) the difference in surface to volume ratio that, in our case, is higher with the particle diameters being in the nano range, while the particles diameter described by Montero et al. [55] measured was of few micrometers (3.3–6.8 µm). 

## 3. Materials and Methods

### 3.1. Chemical and Reagents

Whey protein concentrate (WPC) 80 was gently provided by I.T.ALI. (Reggio Emilia, Italy). The protein content was 80% (w/w). *Haematococcus pluvialis* (*H.p.*) powder was obtained from a local supplier. Ethyl acetate, dimethyl sulfoxide (DMSO), 2,2′-azino-bis(3-ethylbenzothiazoline-6-sulphonic acid) (ABTS), potassium persulfate, ascorbic acid, hydrochloric acid, ferric chloride, HPLC-grade acetone, pepsin, pancreatin, trypsin, and sodium cholate were purchased from Sigma-Aldrich (St. Louis, MO, USA). All enzymes were of porcine origin.

### 3.2. Oleoresin Extraction

*H.p.* oleoresin was extracted using the protocol proposed by Bustos-Garza and co-workers with minor modifications [56]. Briefly, dry algae powder was pretreated by mixing 5 g of algae and 1 mL of 3 M HCl and treating the sample in a microwave oven for 1 min at 100 W. The pretreated algae were extracted with 25 mL of ethyl acetate in a tube with a screw cap for 60 min under agitation at 50 °C in a thermal bath. The solid portion was separated by centrifugation at 3000 × *g* for 10 min to eliminate the biomass. The oleoresin was dried by rotary evaporator (Buchi, Switzerland) and kept in the dark at 4 °C until use.

### 3.3. Spectrophotometric Analyses

#### 3.3.1. ASX Quantification

Quantification of ASX was performed using a UV/Vis spectrophotometer Evolution 201 (Thermo Scientific, Waltham, MA, USA). The samples were diluted in ethyl acetate and the absorbance measured at 480 nm. The concentration of ASX was calculated following the equation:

(1) $$\left[A\right]=\frac{10\times {\;A}_{480}\times \;\mathrm{DF}}{E_{\left(1\%;1\mathrm{cm}\right)}\times \;d}$$

where [A] is the concentration of ASX expressed as mg/mL; A_480_ the sample absorbance at 480 nm; DF: dilution factor; E(1%; 1 cm): ASX percent solution extinction coefficient ((g/100 mL)^−1^ cm^−1^) in ethyl acetate (2150); d: the optical path (cm).

#### 3.3.2. Turbidity Analysis

Turbidimetric analysis was performed by monitoring the absorbance at 660 nm [57].

### 3.4. HPLC Analysis

Reversed-phase HPLC of astaxanthin-containing samples was performed with a Beckman System Gold (Beckman Coulter, Brea, CA, USA) on a C30 column (4.6 mm × 250 mm, particle size 5 µm) (YMC Europe, Schermbeck, Germany) following a previously described method [58] with minor modifications. The absorbance was monitored at 480 nm by a Beckman 168 diode array detector. The injection volume was 50 µL. The elution was carried out at a flow rate of 1 mL/min using acetone (solvent A) and water (solvent B) as follows: isocratic elution at 84:16 (A:B) for 10 min and a gradient to 97:3 (A:B) for 100 min.

### 3.5. H.p. Oleoresin NP Preparation

Astaxanthin nanoparticles (NPs) were produced following the method described previously [27] with some modifications. 

Briefly, WPC was solubilized in distilled water. Different concentrations between 1% and 10% were tested. The solution was stirred for 30 min at room temperature. *H.p.* oleoresin was diluted in ethyl acetate (1%–11% concentration range) and combined with the protein solution at a ratio of 9:1 (protein solution/oleoresin). A fine emulsion was produced using an ultrasonicator for 10 min at a potency of 10 W (Microson ultrasonic cell disruptor XL), differently from Yi et al. [27], that used a combination of high-shear and high-pressure homogenization. At the end of the process, ethyl acetate was removed using nitrogen flow in the dark. The NPs were kept in the dark at 4 °C until use.

### 3.6. Characterization of Astaxanthin Nanoparticles

#### 3.6.1. Dynamic Light Scattering (DLS) Analysis

Z-average (mean diameter), zeta-potential (charge) and polydispersity index (PDI) of NPs were analyzed by dynamic light scattering (DLS) principles using a Malvern Zetasizer (Nano-ZS; Malvern Instruments, Worcestershire, UK) at 25 °C. Prior to the analysis, the samples were diluted 80 times to avoid multiple scattering effects. PDI values ranging from 0 to 1 indicated the distributions of the particle sizes, with a value close to 0 indicating a uniform population of particles and a value close to 1 indicating a wide variety of dimensions among the particle size distribution. Zeta-potential gives important information about particle stability, and for values closer to 0, the system is considered not stable due to the absence of a net charge that can contrast the aggregation process of NPs. 

#### 3.6.2. Encapsulation Efficiency

NPs were treated enzymatically with trypsin (2 mg/mL final concentration) in phosphate buffer pH 7.0 for 4 h at 37 °C in a thermoshaker (Biosan, Riga, Latvia). The enzymatically digested solution was mixed with a double volume of ethyl acetate and placed on a rotating shaker for 60 min. The solution was centrifuged at 12,000 × *g* for 5 min to allow the separation of the two immiscible phases. ASX was recovered from the upper phase, diluted opportunely, and quantified by spectrophotometry as described above. The efficiency of encapsulation (EE%) was estimated by the following formula:

(2) $$\mathrm{EE}\%=\;\frac{\mathrm{ASXf}\;}{\mathrm{ASXi}}\times 100$$

where ASXi represents the initial amount of ASX loaded in the NPs and ASXf refers to the amount of ASX extracted from NPs after the enzymatic degradation of the protein shell.

Surface ASX (ASXs) was calculated as follows: 0.5 mL of NPs was mixed with 1 mL of ethyl acetate for 5 min. After centrifugation at 12,000 × *g* for 5 min, the supernatant was analyzed by spectrophotometry to measure ASXs by the following formula:

(3) $$\mathrm{ASXs}\%=\;\frac{\mathrm{ASXs}\;}{\mathrm{ASXi}}\times 100$$

#### 3.6.3. ABTS Radical Scavenging Activity (RSA)

For the ABTS assay, the procedure proposed by Thaipong et al. [59] with some modifications was followed. Two stock solutions of 7.4 mM ABTS and 2.6 mM potassium persulfate were prepared. The working solution was then obtained by mixing the two stock solutions in equal quantities and allowing them to react for 12 h at room temperature in the dark. The solution was then diluted opportunely with methanol (for the *H.p.* oleoresin), or PBS (for ASX NPs) to obtain an absorbance of 0.75 units at 734 nm. Fresh ABTS solution was prepared for each assay. Samples (20 μL) were loaded in a 96-well plate and left to react with 180 μL of the ABTS solution for 2 h in the dark. Then the absorbance was taken at 734 nm using a microplate reader (Bio-Tek, Winooski, VT, USA). Results were presented as % scavenging activity following the equation:

(4) $$\%\mathrm{RSA}=\frac{A\;\mathrm{blank}-A\;\mathrm{sample}}{A\;\mathrm{blank}}\times 100$$

where A blank is the absorbance given by the solvent at 734 nm while A sample is the absorbance given by the sample.

### 3.7. Stability of the NPs

#### 3.7.1. Effect of pH

The stability of NPs was tested in solutions with different pH values (1–10) as adjusted using NaOH or HCl. The stability was evaluated by spectrophotometry reading the absorbance at 660 nm as a turbidimetric index.

#### 3.7.2. UV Irradiation

The stability against UV-B light of NPs and *H.p.* oleoresin (solubilized in DMSO as described above) was studied using a transilluminator (Bio-Rad, Hercules, CA, USA). During the exposure, aliquots of the samples were taken at different time points: 5’, 30’, 60’, and 120’. The % of residual ASX was determined by spectrophotometry as previously described. 

#### 3.7.3. Fe(III)-Induced Oxidation

The stability of encapsulated ASX against the oxidation induced by Fe(III) was analyzed according to the method proposed by Pan and et al. [60] with some modifications. Briefly, NPs were dispersed directly in distilled water at 7.5 µg/mL concentration. *H.p.* oleoresin was solubilized in DMSO and diluted in water to reach the same concentration. The samples (980 µL) were mixed with 10 µL of a FeCl_3_ solution (350 µg/mL) and incubated at room temperature for 24 h. At different time points (20’, 60’, 3 h, and 24 h), an aliquot was taken and ASX quantified by spectrophotometry. The residual ASX from NPs was analyzed after enzymatic digestion with trypsin as previously described. The samples containing *H.p.* oleoresin were directly extracted with two volumes of ethyl acetate. Ascorbic acid (10 µL from a 1.3 mg/mL stock solution in water) was added to the samples before extraction in order to stop the oxidative reactions. 

The % of ASX retained was evaluated using the following equation:

(5) $$\mathrm{ASX}\%=\;\frac{\mathrm{ASXr}\;}{\mathrm{ASXi}}\times 100$$

where ASXr corresponds to the amount of ASX retained after the exposure to the oxidative conditions and ASXi is the initial amount of ASX determined through enzymatic extraction after encapsulation process.

#### 3.7.4. Thermal Treatment

Storage stability of WPC ASX NPs and *H.p.* oleoresin was evaluated by an accelerated system at 65 °C. The samples were kept in a static oven (Memmert, Schwabach, Germany) in closed polypropylene tubes. At different time points, the samples were analyzed for residual ASX content by spectrophotometry. 

### 3.8. Simulated In Vitro Digestion

In Vitro simulated digestion was performed following Infogest protocol [61]. Simulated gastric fluids (SGF) and simulated intestinal fluid (SIF) were prepared following the protocol. The duration of the two phases were 1 h for the gastric and 4 h for the intestinal phase. The enzymes used were porcine pepsin and pancreatin for the gastric phase (pH 3) and the intestinal phase (pH 7), respectively. Bile salt in the form of sodium cholate was added to SIF to a final concentration of 10 mM. Briefly, 0.5 mL of the NPs preparation and SGF were mixed in a 1:1 ratio, and adjusted to pH 3 with 1 M HCl solution. After 1 h, SIF was added at a 1:1 ratio and the solution was adjusted to pH 7 with 1 M NaOH solution. The reaction was conducted at 37 °C in a rotating shaker. Samples were collected every 30 min and extracted with a double volume of ethyl acetate. The amount of ASX released from WPC ASX NPs were evaluated by spectrophotometry. De-esterification degree of ASX was evaluated through HPLC analysis.

#### Statistical Analysis

All measurements were performed in triplicate and the results were reported as mean value ± standard deviation. Statistical analysis was performed using one-way analysis of variance (ANOVA) using SigmaPlot v12.5 software (Systat Software Inc., San Jose, CA, USA).

## 4. Conclusions

In the present work, ASX oleoresin obtained from the microalgae *H.p.* was successfully nanoencapsulated through emulsification–solvent evaporation technique using a perfectly safe and biodegradable protein carrier, providing full characterization of the produced nanoparticles. The obtained results represent the best compromise between the lowest achievable diameter, the highest stability, and the greatest oleoresin load. In particular, besides scarce solubility at the pH range of 3.5–5.5, the NPs obtained showed higher stability toward UV light, metal-induced oxidation, and heat degradation compared to the oleoresin. Simulated digestion of NPs showed a high ASX bioaccessibility (76%) with 75% conversion into the most bioavailable free form. The formulation can be considered as a promising candidate for the delivery of astaxanthin in human and animal diet integration.

## Figures and Tables

**Table 1:** ABTS radical scavenging activity of *H.p.* oleoresin and NPs.

Sample	ASX Concentration (mg/mL)	RSA (%)
*H.p.* oleoresin	0.2	72.1
NPs	0.025	95.8

## Supplementary Materials

The following are available online at https://www.mdpi.com/1660-3397/17/11/627/s1.
