# Supplementary material for: Development and Characterization of Astaxanthin-Containing Whey Protein-Based Nanoparticles
Source: Mar Drugs. 2019 Nov 4;17(11):627. doi: 10.3390/md17110627 (PMC6891650; doi:10.3390/md17110627)

DLS analysis of the NPs obtained with 1% WPC and 4.5% of *H.p.* oleoresin. The percentage distributions are reported by number (**A**), intensity (**B**), and volume (**C**).

Particles with diameter around 1400 nm in B and C are probably due to the presence of dust and not dependent on the encapsulation process.

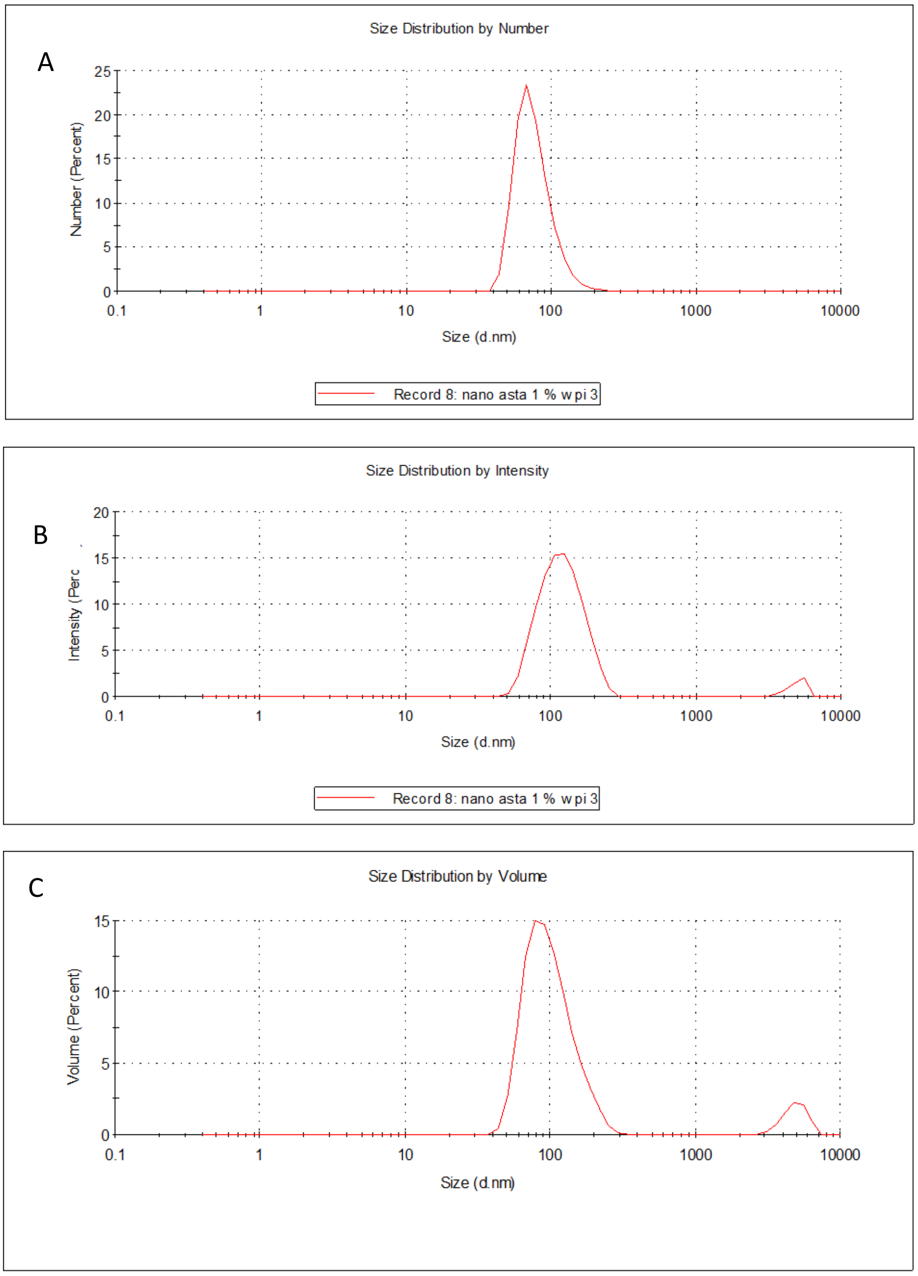

Supplement: Supplementary file 1 [file marinedrugs-17-00627-s001.pdf]
